# Functional Characterization of Class I Trehalose Biosynthesis Genes in *Physcomitrella patens*

**DOI:** 10.3389/fpls.2019.01694

**Published:** 2020-01-20

**Authors:** Tran Le Cong Huyen Bao Phan, Ines Delorge, Nelson Avonce, Patrick Van Dijck

**Affiliations:** ^1^ VIB-KU Leuven Center for Microbiology, VIB, Leuven, Belgium; ^2^ Laboratory of Molecular Cell Biology, Institute of Botany and Microbiology, KU Leuven, Leuven, Belgium; ^3^ Department of Biology, College of Natural Sciences, Cantho University, Cantho, Vietnam; ^4^ Centro de Investigación en Dinámica Celular, Instituto de Investigación en Ciencias Básicas y Aplicadas, Universidad Autónoma del Estado de Morelos, Cuernavaca, Mexico

**Keywords:** *Physcomitrella patens*, trehalose metabolism, PpTPS1, PpTPS2, protonema, gametophytes, sugar signaling, plant hormones

## Abstract

The function of trehalose metabolism in plants during growth and development has been extensively studied, mostly in the eudicot *Arabidopsis thaliana*. So far, however, not much is known about trehalose metabolism in the moss *Physcomitrella patens*. Here, we show that in *P. patens*, two active trehalose-6-phosphate synthase enzymes exist, PpTPS1 and PpTPS2. Expression of both enzymes in *Saccharomyces cerevisiae* can complement the glucose-growth defect of the yeast *tps1∆* mutant. Truncation of N-terminal extension in PpTPS1 and PpTPS2 resulted in higher TPS activity and high trehalose levels, upon expression in yeast. *Physcomitrella* knockout plants were generated and analyzed in various conditions to functionally characterize these proteins. *tps1∆* and *tps2∆* knockouts displayed a lower amount of caulonema filaments and were significantly reduced in size of gametophores as compared to the wild type. These phenotypes were more pronounced in the *tps1∆ tps2∆* mutant. Caulonema formation is induced by factors such as high energy and auxins. Only high amounts of supplied energy were able to induce caulonema filaments in the *tps1∆ tps2∆* mutant. Furthermore, this mutant was less sensitive to auxins as NAA-induced caulonema development was arrested in the *tps1∆ tps2∆* mutant. In contrast, formation of caulonema filaments is repressed by cytokinins. This effect was more severe in the *tps1∆* and *tps1∆ tps2∆* mutants. Our results demonstrate that PpTPS1 and PpTPS2 are essential for sensing and signaling sugars and plant hormones to monitor the balance between caulonema and chloronema development.

## Introduction

Trehalose is a non-reducing sugar consisting of two glucose units in an α,α-1,1 configuration. It is now well known that highly accumulated trehalose in the so-called anhydrobiotic organisms, including yeasts, tardigrades, worms, and some specific plants such as the desert plant *Selaginella lepidophylla,* the resurrection plant *Myrothamnus flabellifolius*, and the grass *Sporobolus* spp, helps them survive during extreme dehydration, heat, or oxidative stress (Adams et al., 1990; Müller et al., 1995; Rebecchi et al., 2009; Wharton, 2011; Vilaça et al., 2012). In plants, trehalose is synthesized through two consecutive enzymatic reactions. The first step is mediated by trehalose-6-phosphate synthase (TPS), which results in the intermediate trehalose-6-phosphate (Tre-6P). A subsequent step results in the formation of the end product, trehalose, catalyzed by trehalose-6-phosphate phosphatase (TPP) (Cabib and Leloir, 1958).

The plant trehalose biosynthesis genes are grouped in three distinct subfamilies according to their similarity to the yeast homologues *TPS1* and *TPS2* in *Saccharomyces cerevisiae* (Leyman et al., 2001; Avonce et al., 2006). The yeast *ScTPS1* gene encodes the TPS enzyme while *ScTPS2* encodes the TPP enzyme (Bonini et al., 2004). In *A. thaliana*, class I TPS enzymes display the highest similarity to ScTps1 and contain four proteins (AtTPS1 – AtTPS4), with AtTPS1, AtTPS2, and AtTPS4 showing TPS activity (Blázquez et al., 1998; Van Dijck et al., 2002; Vandesteene et al., 2010; Delorge et al., 2015). The class II TPS proteins (AtTPS5–AtTPS11) are most similar to ScTps2, but they do not show any detectable TPS or TPP activity upon expression in yeast (Vogel et al., 2001; Ramon et al., 2009). The class III TPP proteins (AtTPPA – AtTPPJ) are smaller isoforms with a well-conserved TPP domain harboring the three L-2-haloacid dehydrogenase (HAD) motifs, and they all function as active trehalose-6-phosphate phosphatases (Vogel et al., 1998; Vandesteene et al., 2012). There are no orthologues of TPP proteins present in *S. cerevisiae*. Interestingly, phylogenetic analysis revealed that TPP proteins are closely related to genes present in *Mycobacterium*, indicating that the class III genes seem to be of bacterial origin (Avonce et al., 2006; Avonce et al., 2010).

Tre-6P acts as a sugar-signaling metabolite that regulates plant metabolism and affects many aspects of plant growth and development (Schluepmann et al., 2004; Lunn et al., 2006; Wahl et al., 2013; Lunn et al., 2014; Bledsoe et al., 2017). Tre-6P is considered as a specific sensor for sucrose availability. Upon carbon starvation Tre-6P levels were low in *A. thaliana* seedlings and they increase strongly by addition of sucrose (Lunn et al., 2006). There is a positive correlation between Tre-6P and sucrose levels under various growth conditions. Many studies have confirmed this correlation in *A. thaliana* rosettes (Lunn et al., 2006; Wingler et al., 2012; Carillo et al., 2013; Yadav et al., 2014), seedlings (Lunn et al., 2006; Yadav et al., 2014), developing seeds (Thiel et al., 2011), and shoot apex (Wahl et al., 2013), as well as in wheat grains (Martínez-Barajas et al., 2011), and developing potato tubers (Debast et al., 2011). Small changes in Tre-6P levels, as a result of increased or decreased gene expression/enzyme activity, can perturb metabolic signaling, leading to reprogramming of expression of hundreds of genes involved in growth and stress responses (Griffiths et al., 2016). The tight regulation of sucrose levels by Tre-6P might be a homeostatic mechanism of the plant ensuring levels of sucrose to be contained in a certain range, optimal for growth (Lunn et al., 2014). Tre-6P also stimulates starch synthesis by promoting thioredoxin-mediated AGPase redox activation (Kolbe et al., 2005), and promotes biosynthetic processes in seedlings in response to high levels of sucrose (Delatte et al., 2011). SNF1-related protein kinase 1 (SnRK1), which is known to inhibit plant growth (Baena-González et al., 2007), is inhibited by Tre-6P (Zhang et al., 2009) and thereby promotes survival of growing tissues of *Arabidopsis* under stress. Moreover, carbon utilization and carbon allocation over plant growth are regulated through the SnRK1 signaling pathway involving Tre-6P, SnRK1, and the sugar-regulated transcription factor basic region leucine zipper transcription factor11 (bZIP11) (Delatte et al., 2011). Tre-6P signaling plays a crucial role in regulation of floral transition in *A. thaliana* (Wahl et al., 2013) and is involved in stress responses. Overexpression of rice *TPP1* in developing maize ears with reduced Tre-6P amounts led to an increase of crop yield in non-drought and mild or even severe drought conditions (Nuccio et al., 2015).

In most vascular plants, only minute amounts of trehalose are detected, which implies that trehalose could not act as an osmoprotectant during stress conditions, as seen in stress-resistant species such as *Selaginella lepidophylla*, where trehalose accumulates to high amounts (Vogel et al., 2001; Fernandez et al., 2010; Pampurova et al., 2014). Therefore, several initiatives were taken to increase trehalose levels by expressing microbial *TPS* and *TPP* genes in plants. Such transgenic model and crop plants showed improved stress tolerance, but unfortunately, also resulted in abnormal plant development. Transgenic plants experienced stunted growth, abnormally shaped leaves, delayed flowering, and many other phenotypic effects (Romero et al., 1997; Penna, 2003; Ge et al., 2008; Li et al., 2011; Delorge et al., 2014). These results suggested that trehalose metabolism and plant growth signaling pathways interfere with each other.

*Physcomitrella patens* is an important model plant in genetic studies due to numerous advantages such as a dominant haploid phase of the life cycle, which allows phenotype selection upon gene modification. Protoplasts can easily be isolated from gametophytes and regenerate directly into filamentous tissues. Furthermore, efficient homologous recombination allows precise inactivation or modification of genes (Cove, 2005). *P. patens* has been widely used as a model organism in fundamental research, developmental studies, and as a tool to produce biopharmaceuticals on a large scale (Cove et al., 1991; Baur et al., 2005; Weise et al., 2007; Reski et al., 2015).

In *P. patens* there are two alternative generations: the haploid gametophyte stage and the diploid sporophyte stage. The protonema filaments of the gametophyte are generated by spore germination. The protonema expands its filaments to form a colony comprising chloronema and caulonema. However, at first protonema only consists of chloronemal cells, which are densely packed with many chloroplasts and divide more slowly. Next, caulonemal cells are differentiated from the chloronemal apical cells. Caulonemal cells contain fewer chloroplasts and, due to their increased rate of cell division, cause rapid colony expansion, allowing the moss to grow (Cove, 2005). The balance between these two types of tissue depends on various factors including light, sugars, and plant hormones (Reski and Abel, 1998; Thelander et al., 2005; Jang and Dolan, 2011). In high-energy conditions, such as high light and increased sugar concentration in the medium, caulonema filaments are induced, whereas low energy, including low light and reduced levels of sugars, stimulates chloronema branching (Thelander et al., 2005).

So far, studies in sugar metabolic signaling, particularly in trehalose metabolism, are limited in *P. patens* (Nagao et al., 2005; Nagao et al., 2006; Rother et al., 2006; Erxleben et al., 2012; Arif et al., 2018). As a bryophyte, *P. patens* represents an ancient lineage of early-diverging land plants and it is an excellent model to study the role of the metabolism of trehalose in plants from an evolutionary perspective. Therefore, in this study, we generated class I TPS knockout plants including *tps1∆, tps2∆*, and *tps1∆ tps2∆* mutants to functionally characterize these proteins, and to determine the role of class I trehalose biosynthesis enzymes in response to various factors in this moss.

## Materials and Methods

### Yeast Strains, Culture Conditions, Transformation, and Complementation Assay

*S. cerevisiae* wild-type strain, W303-1A (Thomas and Rothstein, 1989); *TPS1* deletion strain, YSH290 (W303-1A, *tps1∆*::*TRP1*) (Hohmann et al., 1993); *TPS2* deletion strain, YSH450 (W303-1A, *tps2∆:*:*LEU2*) (Neves et al., 1995); and *TPS1 TPS2* deletion strain, YSH652 (W303-1A, *tps1∆*::*TRP1*, *tps2∆*::*LEU2*) (Neves et al., 1995) were used in this study. Yeast cells were grown at 30°C or at 38°C in synthetic medium with the appropriate auxotrophic requirements and supplemented with 2% (w/v) glucose or 2% (w/v) galactose, as described previously (Blázquez and Gancedo, 1995).

Class I *TPS* genes of *P. patens* were cloned behind the *CUP1* promoter in the yeast expression vector pSAL4 (Zentella et al., 1999) for the complementation assay. Primers used to make these constructs are listed in **Supplementary Table 1**. Plasmids were transformed using the LiAc/PEG method without heat shock (Gietz et al., 1995). Transformants were selected in medium without uracil. Plates were incubated at 30°C (for TPS complementation) or at 38°C (for TPP complementation). After 48 h, the growth of the colonies was analyzed. Growth curves were started with start OD600 of 0.05. Growth was monitored by OD600 measurements every 2 h while continuous shaking in the Automated Microbiology Growth Analysis System (Bioscreen C) for 3 days at 30°C or 38°C.

### Plant Material and Culture Conditions

*P. patens* wild-type strain, “Gransden” isolate, collected from Gransden Wood, in Cambridgeshire (supplied by David Cove, University of Leeds) was used throughout the study, and knockout plants were generated in the Gransden background. Protonema or gametophores were grown axenically on agar plates containing sterile BCD medium (Ashton et al., 1979), supplemented with 1 mM CaCl_2_ and with or without 5 mM ammonium tartrate. The growth of chloronema is induced when ammonium tartrate is included, whereas caulonema formation is enhanced when ammonium tartrate is omitted. The agar plates were overlaid with sterile cellophane. Plants were cultivated in continuous light (45 μmol m^-2^s^-1^) at 25°C. Each week, plants were transferred to new plates. Subcultivation was performed by harvesting plants and homogenizing in sterile water using an ULTRA-TURRAX Tube Drive disperser (IKA).

For most assays, small pieces of protonema were cultivated on solid plates containing cellophane layers, supplied with or without exogenous additives (hormones, sugars). Following hormones were tested: BAP (concentrations of 0.1, 1, and 10 μM) and NAA (concentrations of 0.1, 1, and 10 μM). Following sugars were tested: glucose (25 and 150 mM), sucrose (25 and 150 mM). Expression of *PpTPS1* and *PpTPS2* was monitored by quantitative-PCR after 2, 4, 8, and 24 h (see below).

### TPS Activity Measurements

TPS activity was measured by a coupled-enzyme assay as described by Hottiger et al. (1987). Specific activity was expressed as μkat/g protein. Protein concentration was determined by PIERCE™ 660 nm protein assay reagent (Thermo Scientific) and absorbance was measured at 660 nm using a bovine serum albumin (BSA) standard curve as reference.

### Trehalose Determination Upon Expression in Yeast

Trehalose was determined by hydrolysis to glucose with trehalase (extracted from *Humicola grisea*) and was subsequently quantified by the glucose oxidase:peroxidase method, described by Zentella et al. (1999).

### Metabolite Measurements

Tre-6P was extracted with chloroform-methanol and measured by plant extracts by LC-MS/MS (Lunn et al., 2006) with modifications as described in Figueroa et al. (2016). Trehalose was measured enzymatically in the same extracts using a fluorometric assay as described in Carillo et al. (2013).

### Isolation and Cloning of Class I *TPS* Genes

Coding sequence (CDS) of the class I trehalose-6-phosphate synthase genes of *P. patens* was isolated *via* RT-PCR starting from cDNA of wild-type protonema tissues. To determine the sequence of 5'-end of *PpTPS2V6.1* cDNA, the 5' RACE (Rapid amplification of cDNA ends) System was used, according to the manufacturer's instructions (Invitrogen). After cloning in the appropriate vectors, plasmids were prepared for sequencing (VIB Genetic Service Facility, Belgium). Used primers are listed in **Supplementary Table 1**.

### Quantitative-PCR (qPCR) Analysis

RNA samples were extracted with Trizol (Invitrogen), according to the manufacturer's instructions. To synthesize cDNA, 2 µg total RNA was treated with 1 µl DNase (NEB) for 10 min at 37°C. The reaction was then stopped by denaturation at 75°C for 5 min. cDNA was synthesized from the DNase-treated RNA with the Reverse Transcription System (Promega).

Each qPCR reaction consisted of a 5 μl diluted cDNA sample (2 ng) in a mixture of 10 μl Platinum SYBR Green qPCR Supermix (Invitrogen), 0.8 μl primer-mix (10 μM each), and 4.2 μl H2O. The qPCR reactions were performed in a StepOnePlusTM Real-Time PCR System (Applied Biosystems). Amplification was performed according to the following protocol: denaturation step: 95°C, 15 s; annealing step: 58°C, 30 s; extension step: 72°C, 30 s; all steps repeated for 50 cycles. The housekeeping gene used as a reference is *PpACT1*. Used primers are listed in **Supplementary Table 1**.

### Construction of Transgenic Lines

**Figure 1:**
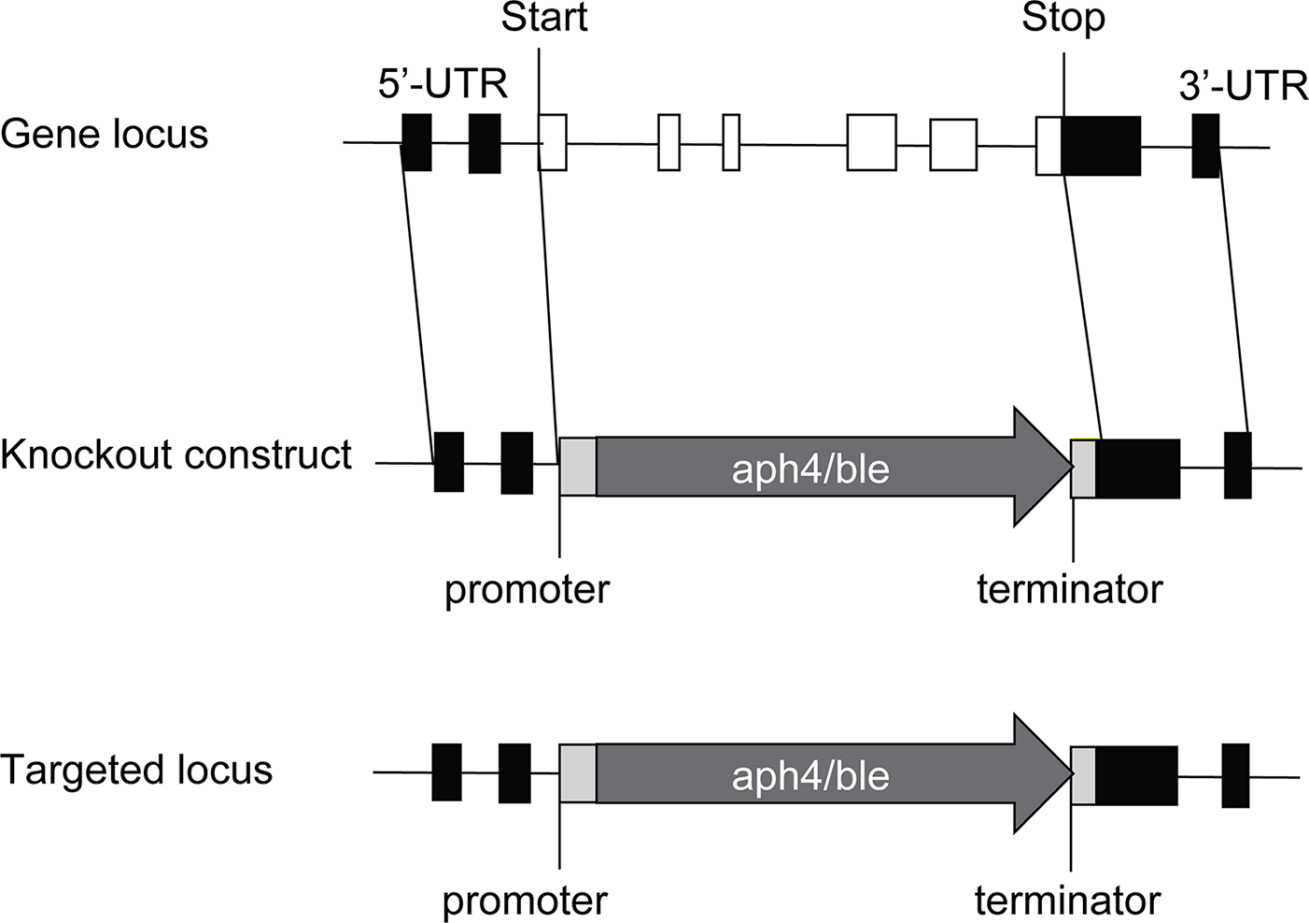
Disruption of *PpTPS1/PpTPS2* genes in *P. patens*. The *PpTPS1* or *PpTPS2* genes are replaced by the antibiotic resistance genes *aph4* or *ble*, respectively. White boxes and black lines between the boxes represent exons and introns, respectively. Black boxes indicate UTR (untranslated region). Light gray boxes indicate promoter and terminator of the resistance gene. The dark gray arrows show the zeocin expression cassette (aph4) or the hygromycin expression cassette (ble).

To completely disrupt the *PpTPS1* or *PpTPS2* gene, approximately 1 kb of flanking genomic regions of 5'-UTR and 3'-UTR of *PpTPS1* or *PpTPS2* was adjacent to the 5'- and 3'-ends of a selection marker in transformation vectors, respectively. Homologous recombination allows efficient integration of the selection cassette at the targeted genomic site, causing a full deletion of the *PpTPS1* or *PpTPS2* gene (**Figure 1**). *PpTPS1* knockout constructs were generated in the pTN186 transformation vector (NIBB, kindly provided by Yukiko Kabeya from the Hasebe laboratory, Japan) that contains the *aph4* gene for selection on hygromycin. *PpTPS2* knockout constructs were made in pHIZ2 (NIBB, kindly provided by Yukiko Kabeya from the Hasebe laboratory, Japan), a plasmid that contains the *ble* gene, allowing selection on zeocin. The final transformation constructs were linearized by restriction digestion prior to PEG-based transformation in protoplasts. Used primers are listed in **Supplementary Table 1**. Furthermore, a double knockout was generated by transformation of the pHIZ2:*TPS2* construct in a verified *tps1Δ* background.

### Isolation of *P. patens* Protoplasts

Five-day-old protonema tissues were digested in 10 ml 8% (w/v) D-mannitol, supplied with 1% driselase (Sigma) for 1–1.5 h at room temperature. Protoplasts were collected *via* filtration on a 100 μm Cell Dissociation Sieve (CD-1TM, Sigma-Aldrich, St. Louis, MO, USA). After several washing steps with 8% (w/v) D-mannitol, protoplast density was determined with a hemocytometer and re-adjusted to an optimal density of 1.6×10^6^/ml.

### Transformation of *P. patens* Protoplasts

Transformation started from 150 μl of protoplast suspension, which was subsequently mixed with 150 μl 2X MMg solution (100 ml contains 6.1 g MgCl_2_.6H_2_O, 8 g D-mannitol, 0.2 g MES, pH 5.6 adjusted with 4M KOH and filter sterilized). DNA (around 60 μg) was quickly added and the whole mixture was transferred to a fresh tube containing 300 μl of 40% PEG solution [10 ml contains 0.236 g Ca(NO_3_)_2_.4H_2_O, 0.047 g HEPES, 0.728 g D-mannitol, 4 g PEG6000, pH 7.5 adjusted with 4M KOH, overnight incubated at room temperature, filter sterilized]. The protoplast mixture was subjected to a 5 min heat shock at 45°C and was afterwards cooled down in a water bath at room temperature. Over the next hour, the transformation samples were step by step diluted with 8% (w/v) D-mannitol to allow full recovery. Finally, protoplasts were left recovering overnight in the dark in PRM-L (BCD medium, 5 mM ammonium tartrate, 6% D-mannitol, 10 mM CaCl_2_). The morning after, protoplasts were centrifuged, resuspended in 9 ml PRM-T [BCD medium, 5 mM ammonium tartrate, 6% mannitol, 10 mM CaCl_2_, 0.4% plant agar (Duchefa)], and plated on cellophane covered PRM-B plates [BCD medium, 5 mM ammonium tartrate, 6% mannitol, 10 mM CaCl_2_, 0.55% Plant agar (Duchefa)]. After 5 days of recovery, protoplasts were transferred to BCDAT plates containing the appropriate antibiotic (25 mgL^-1^ zeocin, Invitrogen or 20 mgL^-1^ hygromycin, Cayla). After 2 weeks of initial selection, plants were transferred to new BCDAT plates without antibiotics in order to avoid unstable transformants. At the end of this period, small parts of the grown protoplasts were again selected on BCDAT plates containing antibiotics. Two weeks of growth will eventually select stable transformants ready for genotyping.

### Phenotypic Analysis

To induce formation of gametophytes, homogenized protonema was cultivated on BCD agar plates, supplemented with 1 mM CaCl_2_ and 5 mM ammonium tartrate (BCDAT medium) for 2 weeks at 25°C under continuous light. Next, small pieces of protonema were transferred to new BCDAT plates. After 1 month, gametophores were ready for phenotyping. Induction of sporophytes occurred in BCD medium, supplemented with 1 mM CaCl_2_ and without ammonium tartrate. Protonema colonies were incubated in baby glass jars containing thick layered agar medium at 25°C in continuous light. After a month, the moss was transferred to a growth chamber adjusted to 15°C under an 8 h light/16 h dark diurnal cycle. Around 14 days later, antheridia and archegonia were formed. To facilitate fertilization, sterile water was added on top of the gametophores. Two weeks later, sporophytes were formed. One month later, matured spores are generated and phenotyping was performed.

To assess the composition of chloronemal cells and caulonemal cells in protonema colonies, protonema filaments were grown on BCDAT plates at 25°C in continuous light. Discrimination of chloronemal and caulonemal cells are based on the angle of the septa. The septa of caulonemal cells are oblique and those of chloronemal cells are perpendicular. The numbers of caulonemal and chloronemal cells in protonema filaments were counted after the cultivation of protonema for 10 days. Growing filaments at the edge of protonema colonies were selected randomly to calculate the percentage of chloronemal and caulonemal cells.

Morphology of the moss was analyzed by a M165C binocular microscope (Leica). Size of protonema colonies, gametophores, and sporophytes were analyzed by ImageJ.

## Results

### *P. patens* Class I TPS Proteins PpTPS1 and PpTPS2 Are Catalytically Active

**Figure 2:**
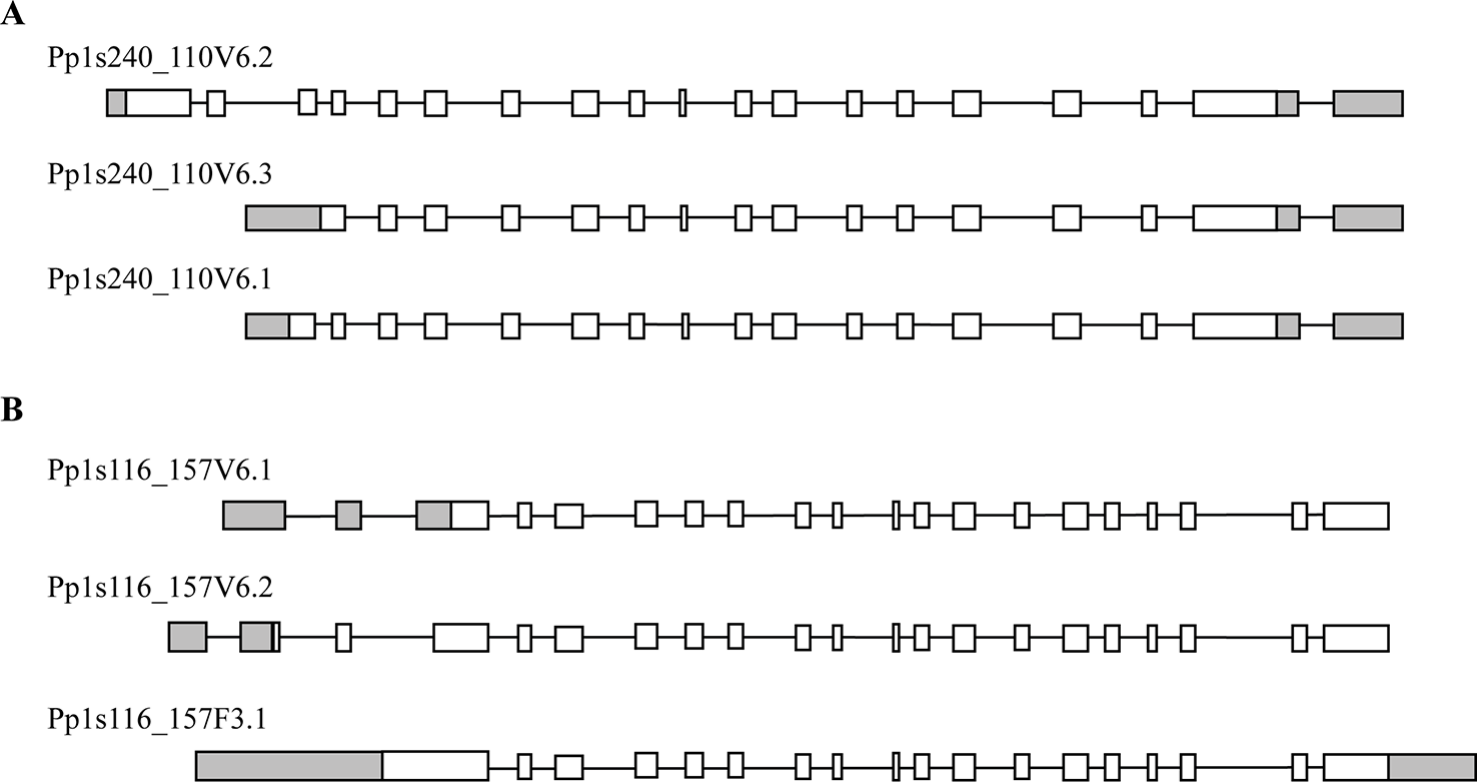
Predicted models by Cosmoss database of the *PpTPS1* **(A)** and *PpTPS2* **(B)** genes. *Pp1s240_110V6.1, Pp1s240_110V6.2, Pp1s240_110V6.3* and *Pp1s116_157V6.1, Pp1s116_157V6.2, Pp1s116_157F3.1* are different splice variants of *PpTPS1* and *PpTPS2*, respectively. White boxes represent exons, black lines indicate introns, and gray boxes show UTR (untranslated region).

Phylogenetic analysis of plant trehalose biosynthesis class I genes showed that two homologues, which are annotated as *PpTPS1* and *PpTPS2*, exist in *P. patens* (Avonce et al., 2010). BLAST analysis in the Genome Browser of Cosmoss database (The *P. patens* genome resource) predicted different splice variants for *PpTPS1* and *PpTPS2* genes. *PpTPS1* has three splice variants, which are *Pp1s240_110V6.1*, *Pp1s240_110V6.2*, and *Pp1s240_110V6.3* (**Figure 2A**). These splice variants mainly differed in the N-terminal end. Similarly, there are three splice variants of *PpTPS2* (*Pp1s116_157F3.1*, *Pp1s116_157V6.1*, and *Pp1s116_157V6.2*) (**Figure 2B**). Alignment analysis with *AtTPS1* elucidated an N-terminal extension in both full length *PpTPS1* (*Pp1s240_110V6.2*) and *PpTPS2* (*Pp1s116_157F3.1*). Interestingly, the splice variant *Pp1s116_157V6.1* (*PpTPS2V6.1*) lacks this N-terminal extension.

**Figure 3:**
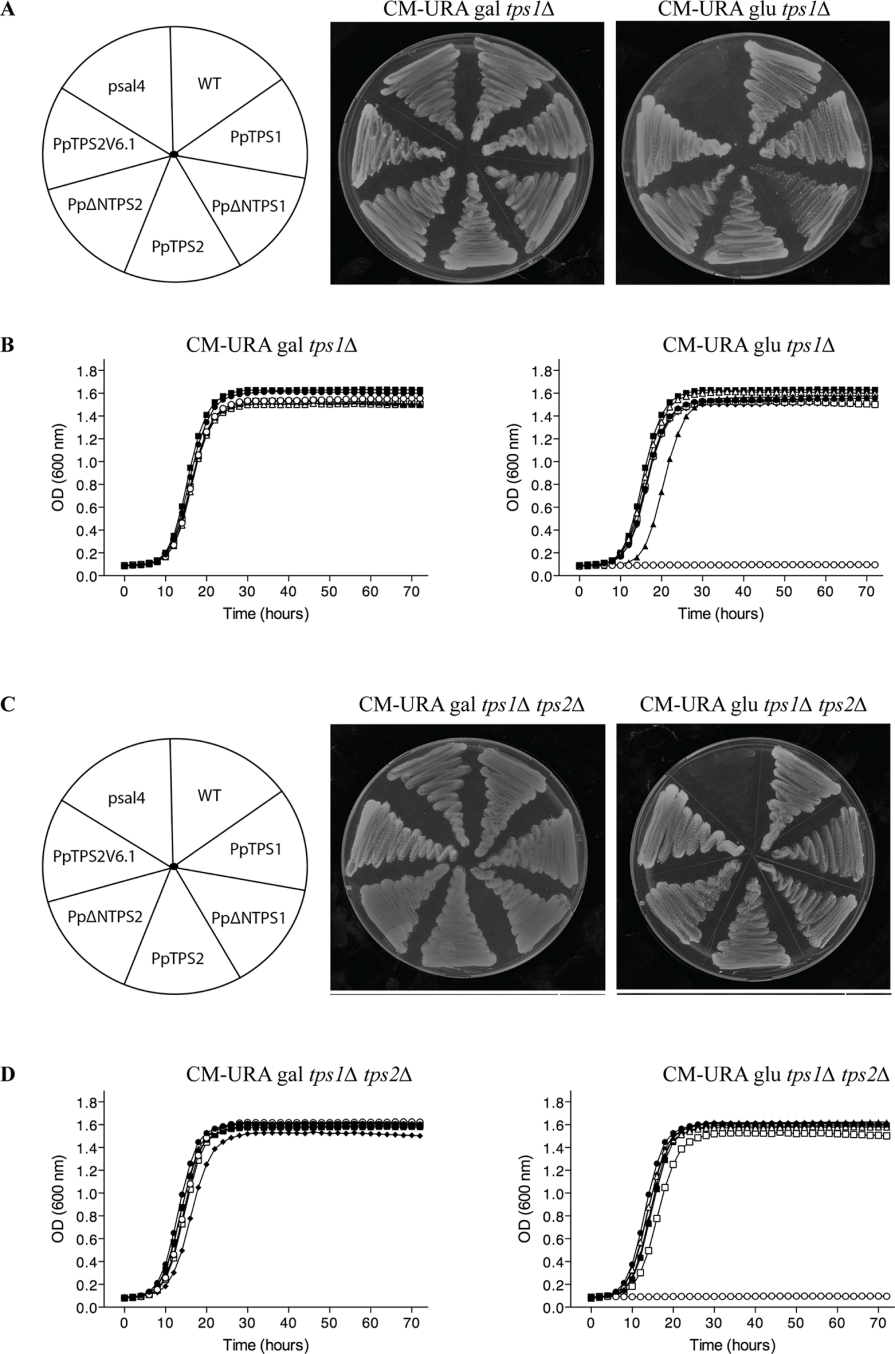
Complementation studies of *PpTPS1*, *PpTPS2*, and *PpTPS2V6.1* in the yeast *tps1∆* **(A** and **B)** and *tps1∆ tps2∆* **(C** and **D)** mutants. **(A** and **C)** Complementation assay was performed on plates with galactose or glucose. Copper sulfate was added to a final concentration of 100 μM to induce expression. The wild-type yeast strain transformed with the empty vector was used as a control (WT). **(B** and **D)** Bioscreen analysis was done in liquid synthetic medium lacking uracil with galactose or glucose and 100 μM copper sulfate. Wild-type (WT) strain transformed with pSAL4 ( ● ), the deletion strain transformed with pSAL4 ( ○ ), or *PpTPS1* ( ▲ ), or *PpTPS2* ( △ ), or *Pp∆NTPS1*( ■ ), or *Pp∆NTPS2* ( □ ), or *PpTPS2V6.1* ( ◆ ).

In order to functionally characterize these two genes, we isolated the coding sequence of class I genes by RT-PCR from protonemal cDNA. Among the three predicted variants of *PpTPS1*, only *Pp1s240_110V6.2* (named as *PpTPS1*) could be isolated. In addition, the splice variant *Pp1s116_157V6.2* (*PpTPS2V6.2*) might be a pseudogene as its sequence contains a premature stop codon and was therefore not included for further analysis. Next, the three variants of class I genes (*PpTPS1*, *PpTPS2*, and *PpTPS2V6.1*) were cloned in a yeast expression shuttle vector, pSAL4, controlled by the *CUP1* promoter. N-terminally truncated versions of *PpTPS1* and *PpTPS2* were also cloned in pSAL4. The first 155 residues at the N-terminal end of PpTPS1 or PpTPS2 were removed, producing the truncated versions of PpTPS1 and PpTPS2. Final constructs were transformed in the yeast *tps1∆* and *tps1∆ tps2∆* mutants to determine whether the class I TPS enzymes can complement the growth defect on glucose-containing medium of the yeast mutants. The yeast *tps1∆* and *tps1∆ tps2∆* mutants are unable to grow on glucose as carbon source, as ScTps1 is required for regulating the flow of glucose into glycolysis (Blázquez et al., 1993; Thevelein and Hohmann, 1995) (**Figure 3**). Apart from plate assays on solid medium, liquid growth assays were performed starting from an initial OD_600_ of 0.05. Both on plates and in liquid media, growth of the yeast *tps1∆* and *tps1∆ tps2∆* mutants was restored on glucose containing medium (**Figure 3**) and therefore all three enzymes are considered as active trehalose-6-phosphate synthases.

**Figure 4:**
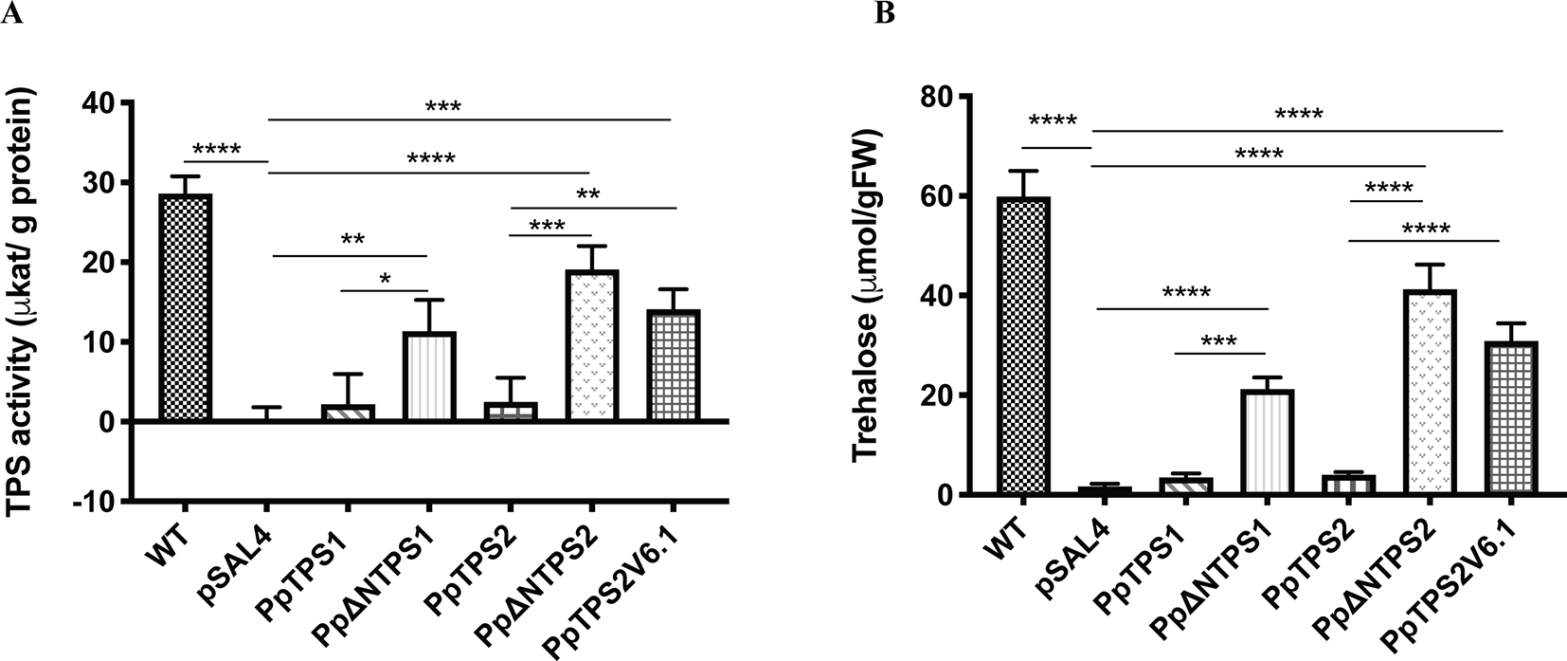
**(A)** TPS activity and **(B)** trehalose measurements in the yeast *tps1Δ* mutant transformed with *PpTPS1*, *PpTPS2*, or *PpTPS2V6.1*. Cells were grown on synthetic medium lacking uracil supplied with 2% galactose. Data represent the mean ± SD of three biological repeats. Statistical analysis with one-way ANOVA, *p ≤ 0.05, **p ≤ 0.01, ***p ≤ 0.001, ****p ≤ 0.0001.

Further evidence supporting the moss class I TPS proteins as active enzymes was provided by measuring TPS activity and trehalose levels upon expression in yeast. The full-length enzymes showed low activity compared to the ScTps1. Truncated PpTPS1 and PpTPS2 had a significantly higher activity, compared to their full-length version (**Figure 4A**). Interestingly, the activity of the splice variant PpTPS2V6.1 was higher than that of PpTPS2 since PpTPS2V6.1 lacks most of the N-terminal extension, which is present in the full length PpTPS2 and which is predicted to exert an inhibitory effect on the enzyme activity. This result is similar to results described by Van Dijck et al. (2002) for *A. thaliana* and for *Ostreococcus tauri* (Avonce et al., 2010). In addition, more trehalose accumulated in the yeast *tps1∆* mutant strains expressing the truncated forms of PpTPS1 and PpTPS2 in comparison with the full-length versions (**Figure 4B**). Likewise, the yeast *tps1∆* containing PpTPS2V6.1 also accumulated more trehalose, which is in accordance with the TPS activity data.

### *P. patens* Class I TPS Proteins Do Not Show TPP Enzymatic Activity

**Figure 5:**
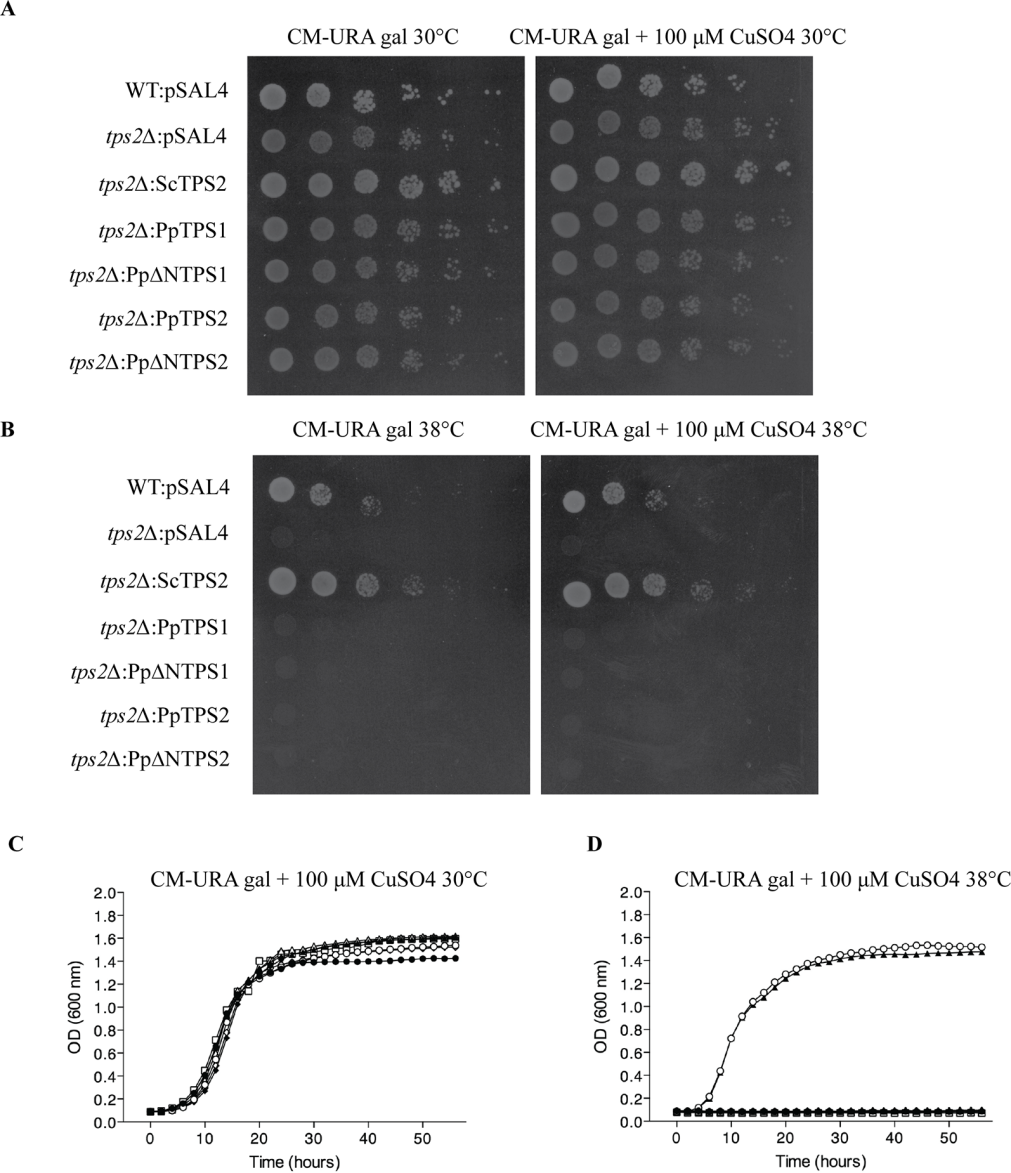
Complementation studies of *PpTPS1* and *PpTPS2* in the yeast *tps2∆* mutant at 30°C or 38°C. Complementation assay was performed on plates supplemented with 2% galactose with or without 100 μM copper sulfate at 30°C **(A)** and 38°C **(B)**. Bioscreen analysis was done at 30°C **(C)** and 38°C **(D)**. Wild-type (WT) strain transformed with pSAL4 ( ▲ ), the deletion strain transformed with *ScTps2* ( ○ ), pSAL4 ( ● ), *PpTPS1* ( △ ), *PpTPS2* ( ◆ ), *PpΔNTPS1* ( □ ), or *PpΔNTPS2* ( ◇ ).

Plant class I TPS proteins harbor a C-terminal part, which is homologous to the TPP domain of *S. cerevisiae* ScTps2 and *E. coli* OtsB (Goddijn and van Dun, 1999). TPP functionality can therefore be tested by complementation studies in the yeast *tps2*Δ mutant. This mutant displays a thermosensitive phenotype since lack of trehalose and accumulating Tre-6P result in a stress-sensitive phenotype at high temperatures. In our hands, none of the class I TPS enzymes of *P. patens* were able to complement the phenotype of the *tps2*Δ mutant at 38°C (**Figure 5**). This result was expected since the TPP domains of these proteins lack the conserved motifs of the phosphatase active site (Leyman et al., 2001; Eastmond et al., 2003; Vandesteene et al., 2010) and none of the previously tested plant class I TPS enzymes have displayed bifunctional enzymatic activity (Avonce et al., 2010).

### Expression Profiling of Class I Trehalose Biosynthesis Genes of *P. patens*

**Figure 6:**
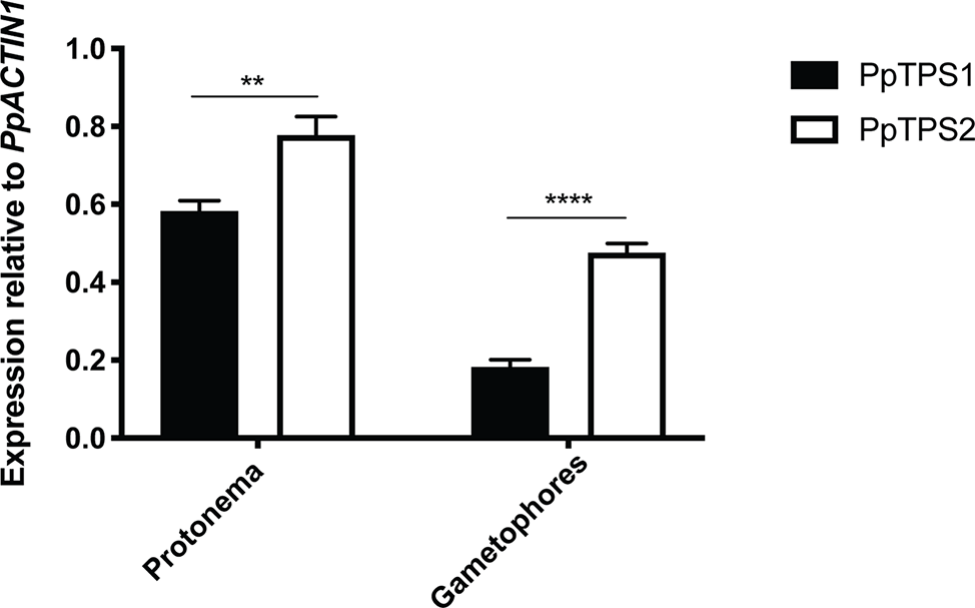
qPCR analysis of *PpTPS1* and *PpTPS2* in different tissues. Expression of *PpTPS1* and *PpTPS2* was measured in protonema and gametophores. Data represent the mean ± SD of three biological repeats after normalization with the housekeeping gene *PpACT1*. Statistical analysis with multiple t tests, **p ≤ 0.01, ****p ≤ 0.0001.

The expression of class I *TPS* genes was analyzed by quantitative-PCR (qPCR). Since no specific primers could be designed for *PpTPS2V6.1*, qPCR analysis for this splice variant was excluded. As shown in **Figure 6**, both *PpTPS1* and *PpTPS2* were expressed in protonema and gametophores. In both tissues *PpTPS2* was expressed at higher levels as compared to *PpTPS1*. In the case of *PpTPS1*, this gene was expressed highly in protonema, whereas its expression was low in gametophores.

### Functional Characterization of Transgenic *P. patens*

**Figure 7:**
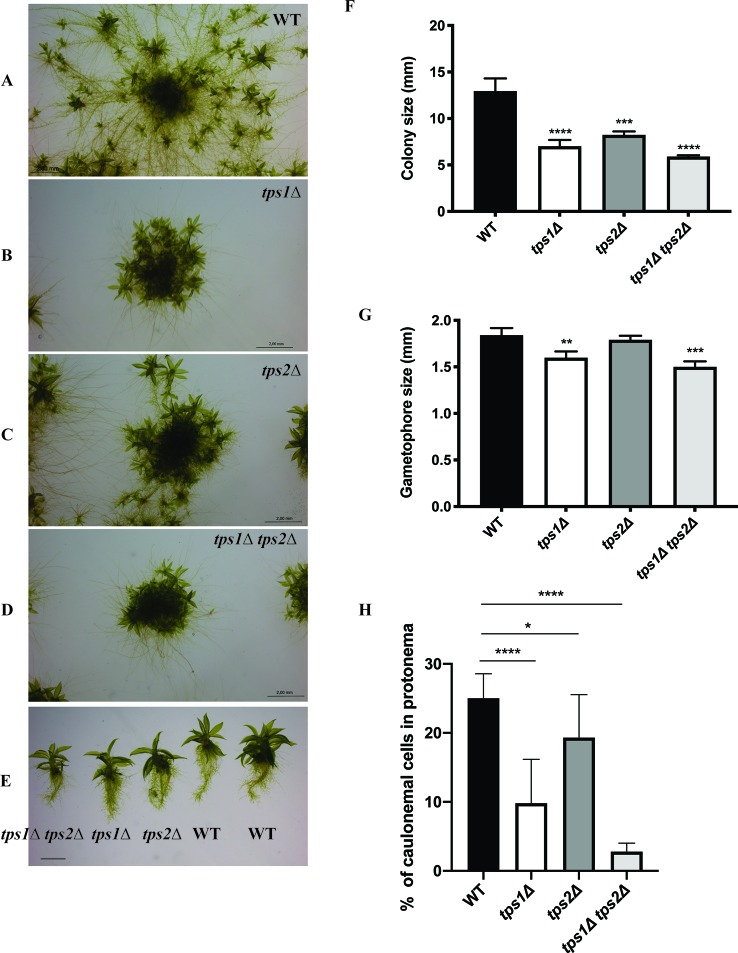
Morphology of protonema of wild-type (WT) and transgenic plants. **(A**–**E)** Representative images of protonema morphology of WT and knockout lines grown on BCDAT medium in continuous light at 25°C for 1 month. Bars represent 2 mm. Measurement of colony size **(F)** and gametophore size **(G)** of WT and transgenic plants. Data represent mean ± SD of three individuals of protonema colonies or gametophores. **(H)** The percentage of caulonemal cells in protonema colonies of WT and mutant lines. Thirteen protonema colonies of each line were selected randomly to count the numbers of caulonemal cells. Statistical analysis with one-way ANOVA, *p ≤ 0.05, **p ≤ 0.01, ***p ≤ 0.001, ****p ≤ 0.0001.

To understand the roles of PpTPS1 and PpTPS2 in plant metabolism and development, transgenic single and double knockout plants (*tps1∆*, *tps2∆*, and *tps1∆ tps2∆* mutants) were generated. Genotyping by PCR was performed to confirm correct deletions. In addition, southern blot analysis was also performed to identify single insertion mutants (**Supplementary Figure 1**). Phenotypic analysis of transgenic plants was performed. A few days after protoplasting, filaments started growing and, after approximately 1 month, plants were ready for subcultivation. In continuous light, moss tends to develop many caulonema filaments in order to stimulate expansion and gametophores are found randomly spread in the plant. In the knockout mutants, caulonema outgrowth seemed to be impaired and gametophores were densely packed together, which might indicate a slower growth rate or altered distribution of energy in the two tissue types, especially in the double mutant as compared to the wild type (**Figures 7A–G**). In addition, the differentiation of chloronema to caulonema was also repressed in the single mutants. This phenomenon was more pronounced in the double *tps1∆ tps2∆* mutant (**Figure 7H** and **Supplementary Figure 2**). These results imply that PpTPS1 and PpTPS2 play an important role in growth and development of *P. patens.* This is similar to *A. thaliana,* where AtTPS1 is essential during embryo development (Eastmond et al., 2002).

**Figure 8:**
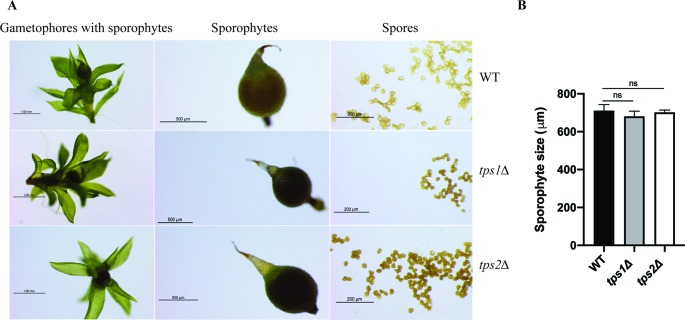
Morphology of sporophytes of wild-type (WT), *tps1Δ*, and *tps2Δ* mutants **(A)**. Measurement of sporophyte size of WT and mutants **(B)**. Data represent mean ± SD of six sporophytes for each line. Statistical analysis with one-way ANOVA, ns, no significant difference.

The effect of class I trehalose biosynthesis proteins on sporophytes was also investigated. Sporulation was initiated by growing the moss plants on BCD agar plates without ammonium tartrate for 1 month under continuous light at 25°C. After that, the plants were grown in the condition of 8 h light/16 h dark light cycle at 15°C for another 4 weeks; sporophytes were formed and ready for analysis. The double mutant (*tps1∆ tps2∆*) failed to produce sporophytes, while single mutants (*tps1∆* and *tps2∆*) developed sporophytes and viable spores. However, no significant difference in sizes of spores was detected between single mutants and wild type (**Figure 8**). The result reveals that PpTPS1 and PpTPS2 are necessary, but redundant, for sporophyte production in *P. patens.*

**Figure 9:**
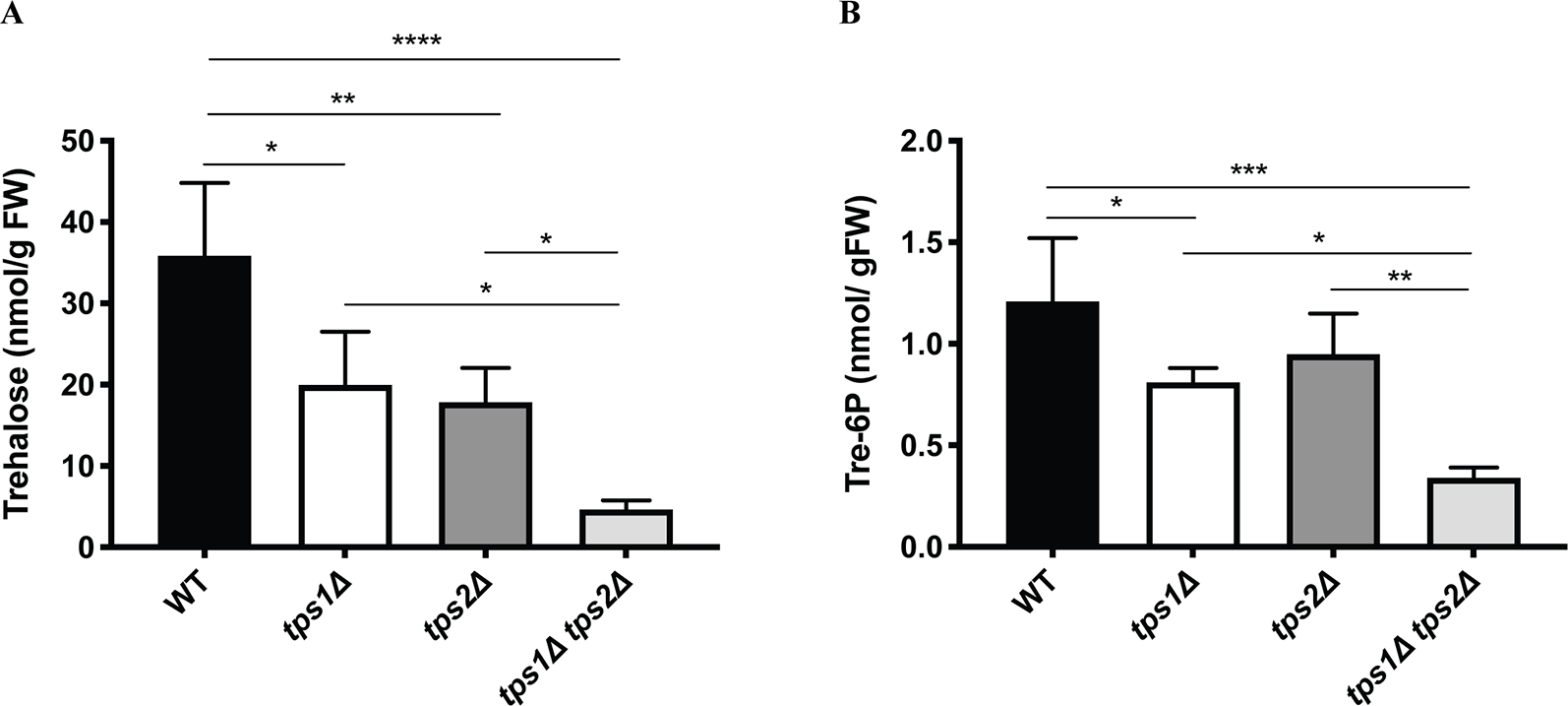
Measurement of trehalose **(A)** and trehalose-6-phosphate **(B)** levels in wild-type (WT) and transgenic plants. Data represent mean ± SD of four biological replicates. Statistical analysis with one-way ANOVA. *p ≤ 0.05, **p ≤ 0.01, ***p ≤ 0.001, ****p ≤ 0.0001.

Next, metabolite levels were determined in the knockout lines to examine whether altered expression levels of class I *TPS* genes affect trehalose and Tre-6P contents. All plants were grown on BCDAT medium for 1 month under continuous light at 25°C. As expected, trehalose levels were significantly reduced in all the knockout lines (**Figure 9A**). Comparison of Tre-6P levels in all knockout lines, compared to the wild type, showed an expected reduction in *tps1∆* and *tps1∆ tps2∆* knockout lines, but no significant reduction in the *tps2∆* line (**Figure 9B**).

### Transgenic Lines in Sugar Growth Conditions

**Figure 10:**
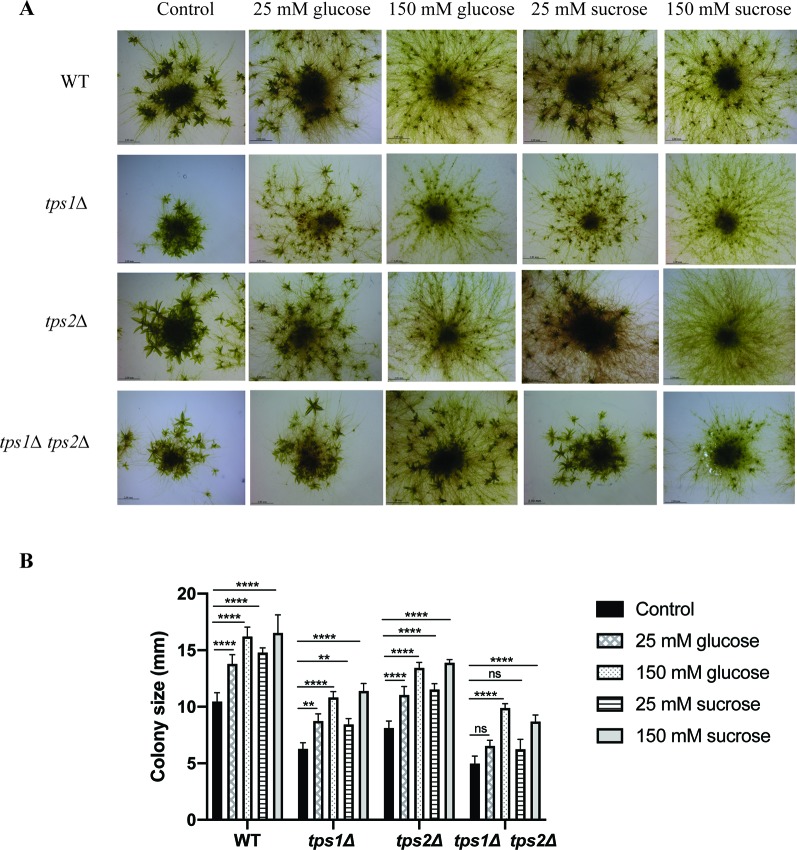
Effect of sugars on growth of wild-type (WT) and transgenic plants. **(A)** Morphology of wild-type (WT) and knockout plants grown on plain BCDAT medium or BCDAT medium supplemented with different sugars (glucose and sucrose). Bars represent 2 mm. **(B)** Measurement of colony size of WT and knockout plants. Data represent mean ± SD of three individuals of protonema colonies. Statistical analysis with two-way ANOVA, **p ≤ 0.01, ****p ≤ 0.0001, ns, no significant difference.

Tre-6P has been reported as a key regulator of carbon utilization for growth and development in plants. Disturbance of Tre-6P levels strongly affects plant metabolic signaling (Eastmond et al., 2002; Schluepmann et al., 2003; Avonce et al., 2004; Schluepmann et al., 2004; Martins et al., 2013; Lunn et al., 2014; Griffiths et al., 2016). In *P. patens* it has been reported that caulonema formation is stimulated in the presence of high levels of sugars or high light, which induce the plant to grow rapidly. In contrast, low light or low levels of sugars will force the plant to produce more energy by formation of photosynthetic tissues, such as chloronema and gametophores (Thelander et al., 2005). Moreover, Olsson et al. (2003) showed that supplied sugars stimulate caulonema filament formation. Therefore, to investigate the relationship of trehalose metabolism and energy availability, we grew the plants on BCDAT agar plates with externally supplied glucose (25 and 150 mM) and sucrose (25 and 150 mM). Addition of glucose or sucrose, even at low levels (25 mM), induced the formation of caulonema in the wild type and in the *tps1Δ* and *tps2Δ* mutants. The double mutant, *tps1∆ tps2∆*, seemed to be less susceptible to the supplied energy (**Figure 10**). When grown on 25 mM glucose, caulonema formation was less induced compared to the wild type and the single mutants. However, increasing the concentration of glucose to 150 mM was able to induce caulonema in the *tps1∆ tps2∆* mutant (**Figure 10**). The double mutant appeared less sensitive to the effect of exogenous sucrose as compared to the effect of glucose.

Besides energy availability, plant hormones also affect many signaling pathways and have pronounced effects on growth and development. The balance between caulonema and chloronema is regulated by hormones. For instance, auxins induce the formation of caulonema, while cytokinins show the opposite effect (Ashton et al., 1979; Reski and Abel, 1985). Thus, we studied the effect of auxin (NAA) and cytokinin (BAP) on trehalose metabolism in *P. patens*. For this purpose, transgenic plants were grown on media containing different concentrations of NAA or BAP, and after 1 month, the effect on morphology was analyzed by microscopy analysis.

**Figure 11:**
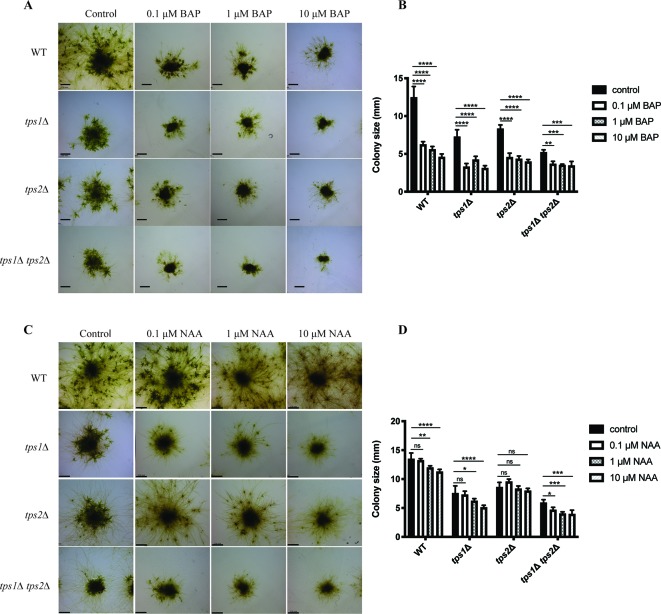
Morphology of wild-type (WT) and transgenic plants grown on different concentrations of cytokinin and auxin. **(A)** Representative images of protonema morphology of plants grown on plain BCDAT medium (control) or BCDAT medium added with different concentrations of BAP (0.1, 1, 10 µM). Bars represent 2 mm. **(B)** Measurement of colony size of WT and mutants after 1-month growth on the medium added with BAP. Data represent mean ± SD of four individuals of protonema colonies. **(C)** Representative of protonema morphology of plants grown on plain BCDAT medium (control) or BCDAT medium added with different concentrations of NAA (0.1, 1, 10 µM). Bars represent 2 mm. **(D)** Measurement of colony size of WT and mutants after 1-month growth on the medium added with NAA. Data represent mean ± SD of four individuals of protonema colonies. Statistical analysis with two-way ANOVA, *p ≤ 0.05, **p ≤ 0.01, ***p ≤ 0.001, ****p ≤ 0.0001, ns, no significant difference.

As expected, BAP reduced the size of colonies and induced the formation of callus-like tissues in the wild type and all mutants (**Figures 11A, B**). This effect was more severe in the *tps1∆* and *tps1∆ tps2∆* mutants. Whereas, NAA-induced caulonema formation was clearly observed in wild-type plants, and in the *tps2∆* line, there was only a very small effect of NAA on the *tps1∆* and *tps1∆ tps2∆* knockout lines as no well-defined outward growth was observed (**Figures 11C, D**).

## Discussion

In this study, we present the molecular and functional characterization of the class I TPS proteins in *P. patens*. By BLAST analysis, using the Cosmoss database, two genes were subsequently found and annotated as *PpTPS1* and *PpTPS2*. By using yeast complementation studies, we show that both genes have TPS enzymatic activity, but no TPP activity, similar to other plant class I enzymes. Moreover, *P. patens* together with *A. thaliana* (Delorge et al., 2015) are the only two plant species described so far with more than one catalytically active TPS enzyme. Interestingly and different from all characterized plants is that *P. patens* has two TPS enzymes with an N-terminal extension. Truncation of the N-terminus of PpTPS1 and PpTPS2 strongly enhanced their enzymatic activity as well as increased trehalose levels when expressed in yeast. Interestingly, a splice variant of PpTPS2 (PpTPS2V6.1) lacking the N-terminus has been predicted in the database. This might be the first time by which such a natural splice variant of the plant class I TPS proteins has been described, suggesting that harboring this variant may allow the plant to modulate rapidly TPS activity and control tightly Tre-6P production in order to respond to environmental stimuli.

Lower trehalose and Tre-6P levels were detected in knockout plants in comparison with the wild type. However, there was no significant reduction in Tre-6P levels in the *tps2∆* line as compared to the wild type. This is possibly due to TPS1, which is still capable of synthesizing Tre-6P and which is still present in the *tps2∆* mutant. Interestingly, Tre-6P levels were significantly lower in the single *tps1∆* mutant, which harbors TPS2. This indicates that TPS1 might be more active than TPS2 as its expression was lower compared to TPS2 (**Figure 6**). Remarkably, low amounts of Tre-6P were still present in the *tps1∆ tps2∆* mutant. This indicates that other active trehalose-6-phosphate synthase(s) might be present in *P. patens*. Four class II TPS proteins (PpTPS3-PpTPS6) were described in *P. patens* (Avonce et al., 2010). Hence, Tre-6P in the *tps1∆ tps2∆* mutant might be synthesized by the class II proteins. Therefore, further research on the functional characterization of class II proteins needs to be performed to confirm this assumption.

Different factors including sugars, light, and plant hormones define the balance between chloronema and caulonema growth in *Physcomitrella*. Conditions such as high light, or the presence of glucose or sucrose that provide energy for plants induce caulonema formation (Thelander et al., 2005). The same was observed in the presence of auxins. Conversely, when conditions are less energy favorable, chloronema branching is stimulated in order to invest in photosynthetic tissues. Furthermore, in the presence of cytokinins, caulonema is inhibited and chloronema is enhanced. It is likely that factors inducing the formation of one type of filaments inhibit the development of the other. Here we demonstrated that caulonema filaments were significantly reduced in knockout lines compared to wild-type plants. Tre-6P was reported to regulate sugar utilization in normal growth (Schluepmann et al., 2003); therefore, a dramatic decrease in Tre-6P levels in the *tps1∆ tps2∆* mutant might lead to a disturbance in carbon allocation, resulting in a reduced growth rate. Additionally, the double mutant was unable to produce sporophytes. Sporophytes largely depend on gametophytes for energy and nutrients. Sporophytes require more energy than they can supply themselves. In *P. patens*, transfer of sugars from gametophores to sporophytes is facilitated through transfer cells, which are present at the boundary of the gametophores and sporophytes (Courtice et al., 1978). It is possible that in the double *tps1∆ tps2∆* mutant the allocation of energy supply towards the sporophytes from gametophores is somehow hampered, leading to a failure in sexual reproduction. We then tested the effect of hormones on plant growth. We show that single knockout lines, especially the *tps1∆* mutant, were less susceptible to NAA-induced caulonema formation. This phenotype was more pronounced in the *tps1∆ tps2∆* mutant, which could explain the lack of caulonema in this mutant. Moreover, it was demonstrated that the transition from chloronema to caulonema is promoted by auxin (Imaizumi et al., 2002; Decker et al., 2006; Jang and Dolan, 2011). We revealed that the differentiation of chloronemal cells to caulonemal cells is significantly decreased in the knockout mutants, especially in the *tps1∆ tps2∆* mutant. These results illustrate that auxin-induced caulonema formation relies on the PpTPS1/PpTPS2-dependent pathway. The link between trehalose metabolism and auxin signaling has also been revealed previously. *A. thaliana* seedlings expressing the *E. coli TPS* gene (*OtsA*) with elevated Tre-6P levels displayed a down-regulation of *auxin/IAA* genes involved in auxin response (Paul et al., 2010). Opposite to auxin, cytokinins did not have a profound effect compared to the effect of NAA, but there was a slight indication towards higher sensitivity in knockout plants. Remarkably, caulonema production in the *tps1Δ* mutant was less than that in the *tps2Δ* mutant under all treatment conditions. Moreover, the former was also less sensitive to sugars and plant hormones than the latter. This suggests that PpTPS1 might be more preferable than PpTPS2 for sensing the growth factors.

Interestingly, an increase in caulonema formation was observed in single and double mutants when high glucose or sucrose was supplied, implying that these plants are still able to sense the supplied energy. Tre-6P is a central metabolic sensor, which regulates plant growth and development. Adequate Tre-6P levels are required for carbon utilization during normal growth. It has been demonstrated previously that *Arabidopsis* plants with reduced Tre-6P levels experienced a growth inhibition when exogenous sugars were added (Schluepmann et al., 2003). This brings us to a question of how energy is sensed in *P. patens*. Sensing energy levels in the cell could be the task of PpSNF1a and/or PpSNF1b (Thelander et al., 2004). A strong connection between trehalose metabolism and SnRK1-signaling has been indicated. SnRK1 is a sucrose-non fermenting related kinase 1, and is part of a serine/threonine kinase family that acts as a metabolite sensor to adapt metabolism accordingly (Jossier et al., 2009). Upon activation by starvation conditions, SnRK1 represses energy-consuming anabolic processes, whereas it induces catabolism, in order to ensure plant survival and stress tolerance. It has been revealed that Tre-6P inhibits SnRK1 (Zhang et al., 2009; Lunn et al., 2014). Recently, it was reported that Tre-6P interacts directly with a catalytic SnRK1α subunit (KIN10) *in vitro* (Zhai et al., 2018). In *P. patens*, PpSNF1a and PpSNF1b have been shown to be involved in the maintenance of sufficient energy levels, clearly demonstrated by the inability of *snf1a∆ snf1b∆* to grow in low light or darkness (Thelander et al., 2004). Interestingly, the phenotype of the *snf1a∆ snf1b∆* mutant is opposite to what is seen in the *tps1∆ tps2∆* mutant. In the former, caulonema formation was highly induced and chloronema branching was reduced, which suggest that the plant experiences a constitutive high energy growth mode. In the *tps1∆ tps2∆* mutant, caulonema filaments were reduced. However, the *tps1∆ tps2∆* mutant was still able to develop caulonema, although to a lesser extent, in high light conditions (**Supplementary Figure 3**). Previously, the link between sucrose and Tre-6P was not fully clear. Nonetheless, it was recently elucidated that Tre-6P regulates sucrose levels by inhibiting the cleavage of sucrose by sucrose synthase (SUS) in castor beans (*Ricinus communis*) (Fedosejevs et al., 2018). This feedback inhibition may control sucrolytic flux from the source to the sink. It was reported that *SUS* expression in potato is dependent on SnRK1 (Purcell et al., 1998). A possibility is that in the *tps1∆ tps2∆* mutant a relief of Tre-6P-mediated SUS repression might occur, in combination with an increase of PpSNF1a and/or PpSNF1b-induced *SUS* expression, leading to changes in hexose levels and altered gene expression. In *A. thaliana*, it was demonstrated that sucrose promotes hypocotyl elongation in the light by activating auxin signaling (Lilley et al., 2012). Recently, sucrose-induced hypocotyl elongation was reported to involve the SnRK1/Tre-6P system as either KIN10 overexpression or a *tps1Δ* mutant shows a defect in hypocotyl elongation, which is induced by sucrose under light/dark cycles (Simon et al., 2018). All taken together, it suggests that Tre-6P/sucrose signaling, PpSNF1, and the auxin pathway might be working together to control growth rate in *P. patens*. The precise mechanism by which external factors and growth regulators affect downstream signaling pathways in *Physcomitrella* needs to be further investigated.

In *S. cerevisiae*, there is a clear connection between trehalose biosynthesis and the activity of hexokinase (Bonini et al., 2003). The *S. cerevisiae tps1Δ* mutant is unable to grow on glucose-containing medium, which is caused by an overactive influx of sugar into glycolysis, mainly due to an overactive hexokinase (Blázquez et al., 1993; Thevelein and Hohmann, 1995). In *A. thaliana*, *AtTPS1* is very lowly expressed in *HXK1*-antisense plants, suggesting that expression of *AtTPS1* is dependent on *HXK1* and that AtTPS1 might participate downstream of HXK1 (Avonce et al., 2004). If this hypothesis occurs in *P. patens*, we might expect similar phenotypes of *hxk1* and class I *TPS* mutants. Indeed, caulonema filaments and size of colonies were reduced in the *hxk1* knockout mutant compared to the wild type (Thelander et al., 2005). Furthermore, the inhibition of cytokinins on caulonema formation was more pronounced in the *hxk1* mutant. These phenotypes are similar to what we observed in the *tps1∆ tps2∆* mutant in our study. Therefore, the class I TPS proteins and hexokinase might work in the same pathway of sensing growth factors to monitor the balance between chloronema and caulonema growth in *Physcomitrella.*

## Conclusions

This work demonstrates the importance of class I TPS proteins in regulating growth and development as well as sexual reproduction of the moss *P. patens*. The growth of caulonema filaments is regulated by PpTPS1 and PpTPS2 as a double knockout mutant displayed a failure in caulonema expansion. Furthermore, the moss is unable to produce sporophytes when the class I *TPS* genes are absent. Additionally, the presence of PpTPS1 and PpTPS2 is essential for sensing and signaling growth factors including sugars and plant hormones. Disruption in Tre-6P production led to a failure in the use of supplied sugar and hormones. These findings are consistent with studies in trehalose metabolism in angiosperms. This indicates that the trehalose metabolism is crucial in regulating plant responses over environmental conditions and that are roles conserved through the plant kingdom. Moreover, it is likely that class I TPS proteins sense energy availability and phytohormones through a pathway involving PpSNF1 and HXK1. Further investigation needs to be performed to support this assumption.

## Data Availability Statement

The raw data and generated strains supporting the conclusions of this article will be made available by the authors, without undue reservation, to any qualified researcher.

## Author Contributions

ID conceived the experiments. ID and TP analyzed the data. NA helped in the generation of moss mutants and contributed to data analysis. TP, ID, and PD wrote the paper. PD supervised the work.

## Funding

This research was supported by the Fund for Scientific Research Flanders (FWO: grant number G.0859.10) and the fund from Vietnam International Education Development (VIED). Ines Delorge was supported by a grant from the Flemish Institute for Science and Technology (IWT).

## Conflict of Interest

The authors declare that the research was conducted in the absence of any commercial or financial relationships that could be construed as a potential conflict of interest.

## Supplementary Material

The Supplementary Material for this article can be found online at: https://www.frontiersin.org/articles/10.3389/fpls.2019.01694/full#supplementary-material

Supplementary Figure 1 Southern blot analysis of tps1∆ mutant (A-C) and tps2∆ mutant (D-F). Genomic DNA of several independent tps1∆ lines (1-5) was digested once by BamHI or XbaI (A), generating a band of approximately 5.2 kb (B) or 4.7 kb (C), respectively. Genomic DNA of several independent tps2∆ lines (1-6) was double digested by KpnI and XhoI or digested once by HindIII (D), generating a band of approximately 7.5 kb (E) or 4.5 kb (F), respectively. A positive control (+) was included to verify correct recognition of the probe. Ladder (L) is shown twice but differently exposed. Arrows show the position of correct digested bands.

Supplementary Figure 2 Caulonemal cells and chloronemal cells in protonema filaments of wild-type (WT) and transgenic plants grown on BCDAT agar medium at 25%°C in continuous light for 10 days. (A) Representative of protonema filaments at the edge of a colony. Magnification 10X. (B) Representative of chloronemal and caulonemal cells. Magnification 40X.

Supplementary Figure 3 Effect of high light (50 µmol m-2s-1) in combination with sugar on growth of wild-type (WT) and the tps1∆ tps2∆ mutant. (A) Morphology of WT and the tps1∆ tps2∆ mutant grown on BCDAT medium, supplied with sugar (either 25 mM glucose or 25 mM sucrose), in high light for one month. (B) Measurement of colony size of WT and the tps1∆ tps2∆ mutant. Data represent mean ± SD of four individuals of protonema colonies. Statistical analysis with two-way ANOVA, *p≤0.05, **p≤0.01, ****p≤0.0001.

Supplementary Table 1 List of primers used in the study. Abbreviations: FW (forward), RV (reverse), GSP (gene-specific primer), CDS (coding sequence), qPCR (quantitative PCR).
