# Supplementary figures and images for: Functional Characterization of Class I Trehalose Biosynthesis Genes in Physcomitrella patens
Source: Front Plant Sci. 2020 Jan 20;10:1694. doi: 10.3389/fpls.2019.01694 (PMC6984353; doi:10.3389/fpls.2019.01694)

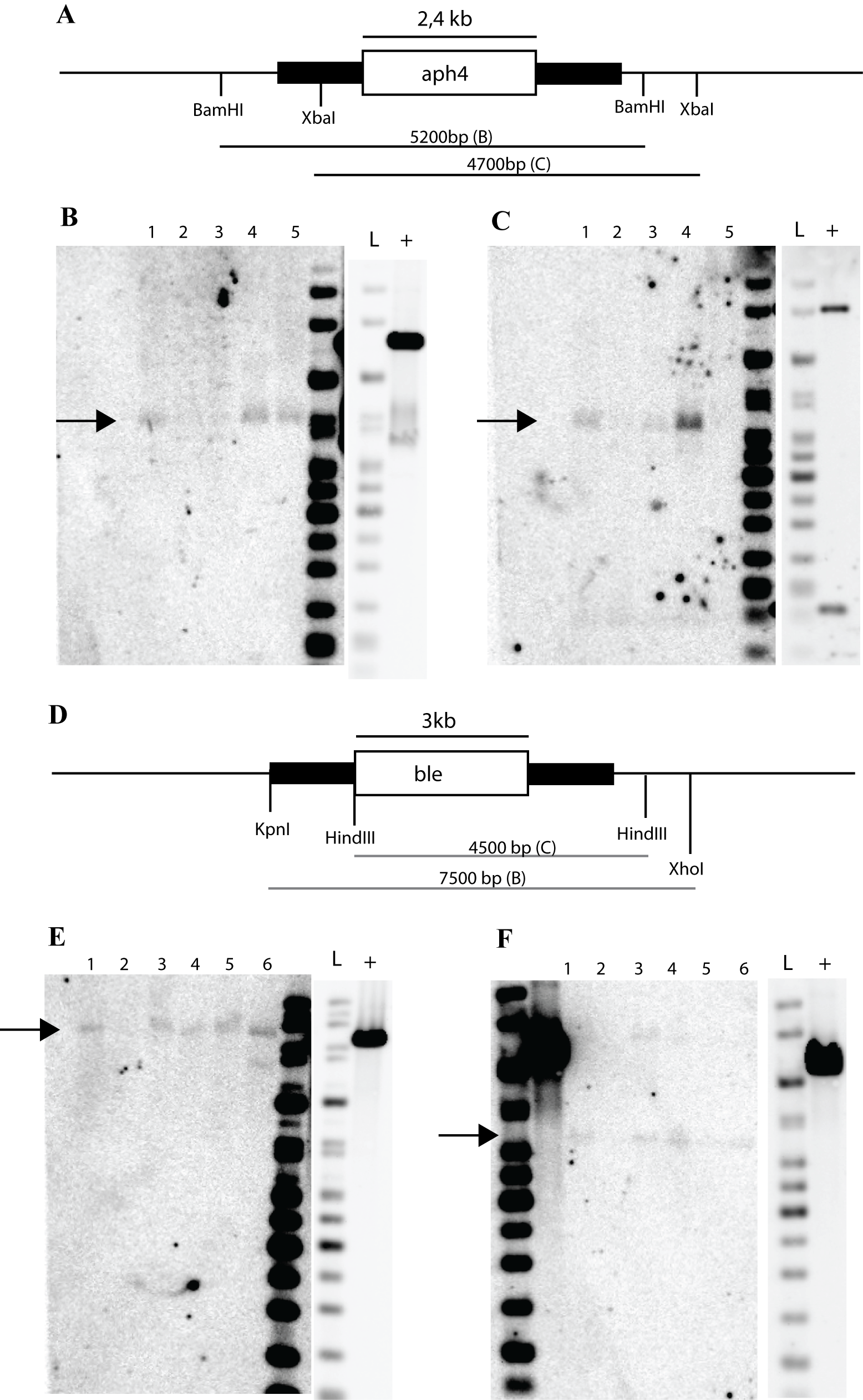

Supplement: Supplementary Figure 1 — Southern blot analysis of tps1∆ mutant (A-C) and tps2∆ mutant (D-F). Genomic DNA of several independent tps1∆ lines (1-5) was digested once by BamHI or XbaI (A), generating a band of approximately 5.2 kb (B) or 4.7 kb (C), respectively. Genomic DNA of several independent tps2∆ lines (1-6) was double digested by KpnI and XhoI or digested once by HindIII (D), generating a band of approximately 7.5 kb (E) or 4.5 kb (F), respectively. A positive control (+) was included to verify correct recognition of the probe. Ladder (L) is shown twice but differently exposed. Arrows show the position of correct digested bands. [file Image_1.tif]

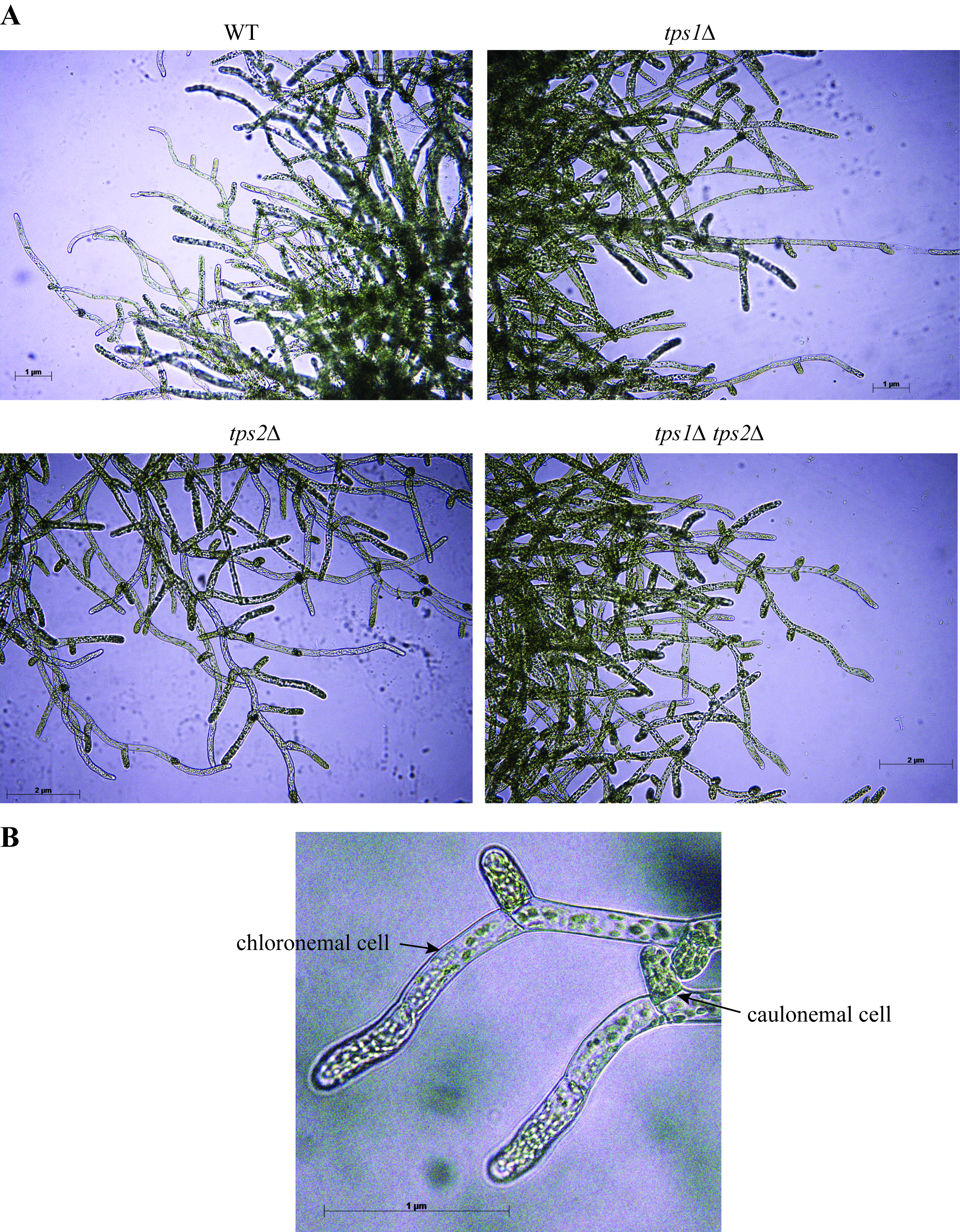

Supplement: Supplementary Figure 2 — Caulonemal cells and chloronemal cells in protonema filaments of wild-type (WT) and transgenic plants grown on BCDAT agar medium at 25%°C in continuous light for 10 days. (A) Representative of protonema filaments at the edge of a colony. Magnification 10X. (B) Representative of chloronemal and caulonemal cells. Magnification 40X. [file Image_2.tif]

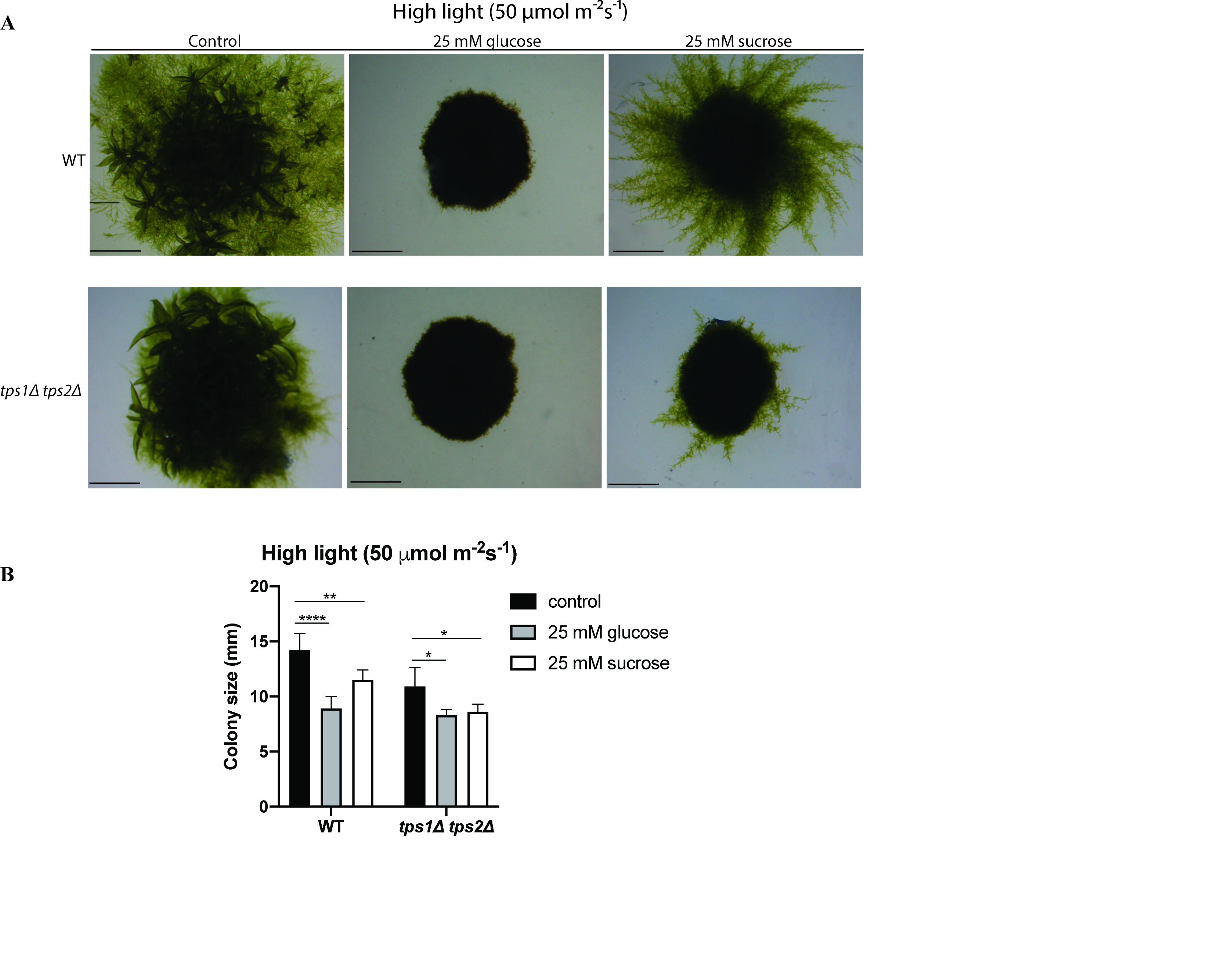

Supplement: Supplementary Figure 3 — Effect of high light (50 µmol m-2s-1) in combination with sugar on growth of wild-type (WT) and the tps1∆ tps2∆ mutant. (A) Morphology of WT and the tps1∆ tps2∆ mutant grown on BCDAT medium, supplied with sugar (either 25 mM glucose or 25 mM sucrose), in high light for one month. (B) Measurement of colony size of WT and the tps1∆ tps2∆ mutant. Data represent mean ± SD of four individuals of protonema colonies. Statistical analysis with two-way ANOVA, *p≤0.05, **p≤0.01, ****p≤0.0001. [file Image_3.tif]
