# Supplementary material for: Functional Characterization of Class I Trehalose Biosynthesis Genes in Physcomitrella patens
Source: Front Plant Sci. 2020 Jan 20;10:1694. doi: 10.3389/fpls.2019.01694 (PMC6984353; doi:10.3389/fpls.2019.01694)
Supplement: Supplementary Table 1 — List of primers used in the study. Abbreviations: FW (forward), RV (reverse), GSP (gene-specific primer), CDS (coding sequence), qPCR (quantitative PCR). [file Table_1.docx]

Supplementary Material

# Supplementary Tables

**Supplementary Table 1.** List of primers used in the study. Abbreviations: FW (forward), RV (reverse), GSP (gene-specific primer), CDS (coding sequence), qPCR (quantitative PCR)

| **Primer** | **Sequence** | **Gene** | **Used for** |
| --- | --- | --- | --- |
| PpTPS1-FW | ATGCCGTACTCTCTGACAACAAAG | *PpTPS1* | isolating CDS of PpTPS1 |
| PpTPS1-RV | TCAACTGACCGATGGCAGCGATTG | *PpTPS1* | isolating CDS of PpTPS1 |
| PpTPS2-FW | ATGCCGTACTCTCTGACAATAAAGGCCTC | *PpTPS2* | isolating CDS of PpTPS2 |
| PpTPS2-RV | TCATAAATCATGCCTAGGGATTCC | *PpTPS2* | isolating CDS of PpTPS2 |
| PpTPS2V6.1-FW | ATGGTTGGCGGCGGCGGGAAC | *PpTPS2V6.1* | isolating CDS of PpTPS2V6.1 |
| PpTPS2V6.1-RV | TCATAAATCATGCCTAGGGATTCCGTTAGG | *PpTPS2V6.1* | isolating CDS of PpTPS2V6.1 |
| PpTPS2-GSP1-RV | ACATGAGGACCAGACCCTGAACCGACC | *PpTPS2V6.1* | 5’ RACE-PCR |
| PpTPS2-GSP2-RV | ACTGCACAGGAGATGTACGTATCTGACG | *PpTPS2V6.1* | 5’ RACE-PCR |
| PpTPS1-pSAL4-FW | CGACTCCTCGAGATGCCGTACTCTCTGACAACAAAG | *PpTPS1* | cloning into pSAL4 |
| PpTPS1-pSAL4-RV | CGACTCGGTACCTCAACTGACCGATGGCAGCGATTG | *PpTPS1* | cloning into pSAL4 |
| PpTPS2-pSAL4-FW | CGACTCGAATTCATGCCGTACTCTCTGACAATAAAGGCCTC | *PpTPS2* | cloning into pSAL4 |
| PpTPS2-pSAL4-RV | CGACTCGGTACCTCATAAATCATGCCTAGGGATTCC | *PpTPS2* | cloning into pSAL4 |
| PpTPS2V6.1-pSAL4-FW | CCGGAATTCGGAATGGTTGGCGGCGGCGGGAAC | *PpTPS2V6.1* | cloning into pSAL4 |
| PpTPS2V6.1-pSAL4-RV | CGGGGTACCTCATAAATCATGCCTAGGGATTCCGTTAGG | *PpTPS2V6.1* | cloning into pSAL4 |
| PpΔNTPS1-pSAL4-FW | CGACTCCTCGAGATGCACCGAAGTGCACAAAGATTACTGG | *PpΔNTPS1* | cloning into pSAL4 |
| PpΔNTPS1-pSAL4-RV | CGACTCGGTACCTCAACTGACCGATGGCAGCGATTG | *PpΔNTPS1* | cloning into pSAL4 |
| PpΔNTPS2-pSAL4-FW | CGACTCGAATTCATGCACCCAAGTAAACAAAGATTACTG | *PpΔNTPS2* | cloning into pSAL4 |
| PpΔNTPS2-pSAL4-RV | CGACTCGGTACCTCATAAATCATGCCTAGGGATTCC | *PpΔNTPS2* | cloning into pSAL4 |
| PpTPS1KO-LB-FW | CGGGGTACCTCTATCAACTTCCTCGTTGTTTCAGC | *PpTPS1* | making knockout transgenic plants (pTN186) |
| PpTPS1KO-LB-RV | CCCAAGCTTACGCCCTCCCTCTGTCAATAATATCTC | *PpTPS1* | making knockout transgenic plants (pTN186) |
| PpTPS1KO-RB-FW | ACATGCATGCCACCGAAGTGCACAAAGATTACTGG | *PpTPS1* | making knockout transgenic plants (pTN186) |
| PpTPS1KO-RB-RV | CGCGGATCCTTGTAATATTGGTCAACTGTATCATCATCTAG | *PpTPS1* | making knockout transgenic plants (pTN186) |
| PpTPS2KO-LB-FW | CGGGGTACCGAATCGTTCTTCTTTCACTTATAGTATCAATTTCC | *PpTPS2* | making knockout transgenic plants (pHIZ2) |
| PpTPS2KO-LB-RV | CCCAAGCTTACGCCTCCCCTCTGTCAATAATATCTC | *PpTPS2* | making knockout transgenic plants (pHIZ2) |
| PpTPS2KO-RB-FW | ACATGCATGCCACCCAAGTAAACAAAGATTACTGGTGG | *PpTPS2* | making knockout transgenic plants (pHIZ2) |
| PpTPS2KO-RB-RV | CGCGGATCCAGCGAGCCGATCCGCCTGAGGC | *PpTPS2* | making knockout transgenic plants (pHIZ2) |
| PpTPS1-qPCR-FW | TGATCTCTCCACCCCGAAAG | *PpTPS1* | qPCR |
| PpTPS1-qPCR-RV | TCCACCCGCGCAGATG | *PpTPS1* | qPCR |
| PpTPS2-qPCR-FW | TCCGGATCATAATGGGTCTCA | *PpTPS2* | qPCR |
| PpTPS2-qPCR-RV | CCGTCTTTGGAGCTGTTCGA | *PpTPS2* | qPCR |
| PpACT1-qPCR-FW | GATAGGAGGGTCTATTTTGGCATCT | *PpACT1* | qPCR |
| PpACT1-qPCR-FW | ATCGTACTCCGACTTCGCAATC | *PpACT1* | qPCR |
